# Combretum Sundaicum in the Cure of the Opium Habit

**Published:** 1908-05-09

**Authors:** 


### COMBRETUM SUNDAICUM IN THE CURE OF THE OPIUM HABIT.

We write this short article, not with any faith in the curability of the opium habit by the drug in question, but in order to draw attention to such facts and statements as have been made about it. It is possible that, knowing of the drug, some of our readers may wish to try it; for as far as can be seen it at least does no harm even if it does no good. The opium habit is usually so refractory to treatment that it is almost impossible to tell whether any particular therapeutic agent is really effective against it. On this account it is right to make extensive trial of any drug that may have seemed to do good in some cases before that drug is lightly passed by as useless.

The history of the discovery of the asserted properties of *Combretum sundaicum* is sufficiently interesting. The news came from Selangor, in the Malay Peninsula, in 1907, that a party of Chinese wood-cutters, when up-country near Seremban, ran short of tea, and tried to make a substitute for it from a jungle plant whose leaves were not too dissimilar to those of the tea plant. They found that an infusion of the sun-dried leaves caused severe diarrhœa, but that when the dried leaves were roasted before being infused no such intestinal symptoms developed. It then happened that the refuse of opium got mixed with some of the combretum leaves, and it was found by those who drank the tea made from the mixture that their habitual desire for opium had disappeared in about a week. The men themselves had no desire to lose their taste for opium in this way.

There is now a liquid extract of the drug obtainable, or an infusion of the roasted leaves may be prepared on the spot. It is apparently better to make the fresh infusion from time to time at short intervals, because it soon ferments when kept. The method of administering the drug is briefly as follows: Two wine-bottles are nearly filled with the decoction of the leaves, and into the first is also put an amount of opium equivalent to the quantity taken by the patient in a given time. An ounce of the mixture is taken several times a day, and after each dose an equivalent quantity of the pure decoction in the second bottle is poured into the first. In this way, as time goes on, the patient receives less and less opium in each dose of the medicine; and when the bottles are finished, a second and a third course are pursued, each time with a smaller quantity of opium in the first of the two bottles. It is said that three sets of bottles-full leave the patient in perfectly good health, and weaned from the opium habit; but so great a result from so simple a procedure certainly sounds too good to be true.

Analyses of the crude plant have been made, by E. F. Harrison in particular, with a view to determining whether any chemical ingredient could be isolated from it, in support of the statement that the drug is so efficacious. So far, however, chemistry has lent no support to the view that the leaves or stem of *Combretum sundaicum* contain any very active principle. It seems clear, at least, that neither an alkaloid nor any crystalline body is present. There is some evidence, however, that the drug contains a glucoside, and when it is remembered how difficult is the chemical investigation of glucoside principles, and yet how potent they may be, as in the case of digitalis, there is as yet no proof that the drug is inert; and, since it seems to cause no harm, it is at least worthy of trial.

